# Combining Spatial Registration With Clutter Filtering for Power-Doppler Imaging in Peripheral Perfusion Applications

**DOI:** 10.1109/TUFFC.2022.3211469

**Published:** 2022-11-24

**Authors:** Michael F. Insana, Bingze Dai, Somaye Babaei, Craig K. Abbey

**Affiliations:** Department of Bioengineering, Beckman Institute for Advanced Science and Technology, University of Illinois at Urbana–Champaign, Urbana, IL 61801 USA; Department of Electrical and Computer Engineering, University of Illinois at Urbana–Champaign, Urbana, IL 61801 USA; Department of Bioengineering, University of Illinois at Urbana–Champaign, Urbana, IL 61801 USA; Department of Psychology, University of California at Santa Barbara, Santa Barbara, CA 93106 USA

**Keywords:** Clutter filtering, motion modeling, phantom studies, power-Doppler ultrasound, scattering model, spatial registration

## Abstract

Power-Doppler ultrasonic (PD-US) imaging is sensitive to echoes from blood cell motion in the microvasculature but generally nonspecific because of difficulties with filtering nonblood-echo sources. We are studying the potential for using PD-US imaging for routine assessments of peripheral blood perfusion without contrast media. The strategy developed is based on an experimentally verified computational model of tissue perfusion that simulates typical in vivo conditions. The model considers directed and diffuse blood perfusion states in a field of moving clutter and noise. A spatial registration method is applied to minimize tissue motion prior to clutter and noise filtering. The results show that in-plane clutter motion is effectively minimized. While out-of-plane motion remains a strong source of clutter-filter leakage, those registration errors are readily minimized by straightforward modification of scanning techniques and spatial averaging.

## I. Introduction

POWER-DOPPLER ultrasonic (PD-US) imaging could become an important tool for monitoring spatiotemporal changes in the muscle perfusion of patients at risk for peripheral arterial disease (PAD) of the lower extremities. PD-US images indicate perfusion qualitatively from the net power within the signal bandwidth that is measured over an ensemble of Doppler pulses [1]. These images faithfully display relative muscle perfusion if the tissue clutter and noise components of the echo signal are minimized to leave a reproducible estimate of blood-echo power. This report describes an analysis of the ultrasonic imaging process with experimentally verified echo simulations that reveal the conditions under which PD-US can reliably represent peripheral muscle perfusion.

PAD is a systemic reduction of capillary perfusion from progressive atherosclerosis occurring across vascular scales [2]. Patient symptoms occur primarily in the extremities and range from muscle cramping to gangrene requiring amputation. PAD patients are also at increased risk for developing coronary artery disease and stroke [2], [3]. As its lifetime occurrence in the US population is 20%–30% [4], advancing PAD poses a significant risk to healthy aging. PAD is often preventable, and some lost perfusion can be recovered if treatment accompanies lifestyle adjustments. Because PAD symptoms are similar to other conditions, there is a growing need for effective techniques that permit frequent monitoring of muscle perfusion in at-risk populations [5].

Ultrasonic imaging of microvascular perfusion without contrast enhancement poses unique challenges for clutter filtering. Blood cell speed in the capillaries is slow (0.1–2.0 mm/s) [6], [7], [8] and directionally diffuse. Conversely, the movement of muscle reflectors relative to the beam axis that occurs from probe jitter, patient breathing, and heart sounds are more spatially coherent. The resting-state motion range of skeletal muscle for fixed-probe scanning (no hand jitter) was found to be routinely small (<0.1 mm) during 2-s Doppler acquisitions [see Fig. 5(a)]. Perfusing blood cells are spatially integrated throughout the moving muscle tissue. Hence, one component of blood-cell motion relative to the sound beam is spatially coherent and coupled to tissue motion, while the second component involves diffuse cell movements within capillaries that are uncoupled to the tissue movements. The latter component is the target of perfusion imaging. The ratio of clutter-echo amplitude to blood-echo amplitude in peripheral perfusion applications is on the order of 5 at 24 MHz, increasing to 16 at 8 MHz.^1^

The high echogenicity, large SNR, and coherent in-plane motion of peripheral muscle scatterers relative to the blood cells enable principal component analysis (PCA) filters to effectively minimize the clutter component [9], [10], [11]. However, if the tissue and blood-cell speeds overlap [18], the separation of clutter and blood singular-value subspaces is incomplete. Some investigators have proposed rigid spatial registration of echo frames before clutter filtering [14], [15], [16], [17], [18]. Registration minimizes any in-plane translation of the dominant clutter echoes between frames, which narrows the clutter singular-value bandwidth and improves the separation between clutter and blood subspaces to enhance PCA filter efficiency. Spatial registration techniques are well described in the ultrasonic imaging literature where the primary applications are 3-D image rendering and multimodal image fusion [19], [20], [21], [22], [23], [24]. This report summarizes our investigation of factors influencing the reliability of PD-US measurements in representing temporal changes in muscle perfusion.

We begin by describing a computational model simulating ultrasonic echo signals from perfused muscle, where scatterers move continuously in three spatial dimensions, while echoes are recorded discretely in scan planes. We also implement a displacement estimator for spatial registration that is applied to echo frames prior to PCA filtering. Simulations provide the exactly known conditions from which errors in spatial registration methods and PD-US signals can be precisely determined with respect to each source contributing to the echo signal. We will show that small out-of-plane tissue movements are the greatest challenge to obtaining consistent PD-US measurements, but there are methods for detecting elevational motion and reducing the uncertainties caused by the losses of interframe echo correlation. Our focus is on simulation and phantom measurement at 5 MHz, but we explore results at 20 MHz that are used for preclinical studies.

## II. Methods

### A. Perfused Tissue Scatterers

We propose a discrete-scatterer model to simulate echo signals from perfused tissues scanned with a linear array transducer. Here, tissues are modeled as a porous matrix composed of random point reflectors that move continuously.

The *n*th scatterer is represented by 3-D Dirac delta *δ*(**x** − **x**_*n*_) at position **x**_*n*_ = (*x_n_*, *y_n_*, *z_n_*)^⊺^ within tissue volume Ω. Scatterers moving relative to the sound beam have positions that are functions of measurement time, **x**_*n*_ = **x**_*n*_(*t*). The scattering field is given by the object function

(1) $$f\left(x,t\right)=\underset{n\in \text{Ω}}{\sum }a_{n}\delta \left(x-x_{n}\left(t\right)\right)$$

where *a*_*n*_ is the reflectivity of the *n*th scatterer. The object may be further parsed into tissue and blood scatterers

(2) $$f\left(x,t\right)=f_{\tau }\left(x,t\right)+f_{\beta }\left(x,t\right)\;=\underset{p\in \text{Ω}}{\sum }\tau \;\delta \left(x-x_{p}\left(t\right)\right)+\underset{q\in \text{Ω}}{\sum }\beta \;\delta \left(x-x_{q}\left(t\right)\right)$$

where *τ* and *β* are the reflectivities of tissue and blood cells, respectively, which are assumed to be constant throughout Ω. The locations of tissue **x**_*p*_ and blood **x**_*q*_ scatterers define the locations of all reflectors **x**_*n*_ ∈ {**x**_*p*_, **x**_*q*_} and *a_n_* = {*τ, β*}.

### B. Echo Signal Model

The signal model converts the 3-D object function from (1), a continuous function of space and time, into a time series of discretely sampled 2-D radio frequency (RF) echo frames. A spatiotemporal object is mapped into each measurement of the dataset via the *spatial sensitivity function* (SSF), *h*_*S*_(**x**). The SSF characterizes contributions from lateral (*x*), elevational (*y*), and axial (*z*) positions relative to the beam axis at a given measurement time. Specifically, for pulse-echo impulse response function, *h*(**x**, *t*), the SSF is *h_S_*(**x**) = *h*(**x**|*t*) [27], [28]. Since scatterers move slowly compared to the acquisition frame rate, we assume that all scatterers are essentially frozen during the time an echo frame is acquired. Also, we model the SSF as unchanged within volume Ω (local shift invariance).

We indicate the fast-time axis by *t_k_* = (*k* − 1)*T_k_*, 1 ≤ *k* ≤ *K*, with fast-time sampling rate 1/*T*_*k*_ [MHz]. It logs the arrival of echo samples along the beam axis at depth *z_k_* = *ct_k_*/2 for sound speed *c* [mm/*μ*s]. Samples in the scan plane along the axis perpendicular to the beam axis are given by *x_ℓ_* = (*ℓ*−1)*X*, where 1 ≤ *ℓ* ≤ *L*. *X* [mm] is the lateral sampling interval as determined by the pitch of the array. The frame-time (or slow-time) axis is *t_m_* = (*m* − 1)*T_m_*, 1 ≤ *m* ≤ *M* at Doppler frame rate 1/*T_m_* [Hz]. From these variables, each beamformed echo signal frame *g_kℓm_* is described for an axial sample *k*, lateral sample *ℓ*, and frame *m* as

(3) $$g_{k\ell m}=\epsilon_{k\ell m}+\int_{x\in \text{Ω}}dx\;h_{S}\left(x-x_{\ell },y,z-Ct_{k}/2\right)\;f\left(x,t_{m}\right)\;=\epsilon_{k\ell m}+\int_{x\in \text{Ω}}dx\;h_{S}\left(x-x_{k\ell }\right)\;f\left(x,t_{m}\right)$$

where **x**_*kℓ*_ = (*x_ℓ_*, 0, *z_k_*)^⊺^ locates echo samples in the (*x, z*) scan plane with acquisition noise *ϵ*. The number of integrals in (3) is determined by the dimension of *d***x** in this case three. We are implicitly assuming that *f*(**x**, *t_m_, t_k_*) = *f*(**x**, *t_m_*) by assuming that the scatterer velocities are much slower than the rate of data acquisition. Simulation parameters are listed in Table I.

Combining (1)–(3), we obtain

(4) $$g_{k\ell m}=\epsilon_{k\ell m}+\underset{n\in \text{Ω}}{\sum }a_{n}h_{S}\left(x_{n}\left(t_{m}\right)-x_{\ell },y_{n}\left(t_{m}\right),z_{n}\left(t_{m}\right)-z_{k}\right)\;=\epsilon_{k\ell m}+\underset{n\in \text{Ω}}{\sum }a_{n}h_{S}\left(x_{n}\left(t_{m}\right)-x_{k\ell }\right)\;=\epsilon_{k\ell m}+\underset{p\in \text{Ω}}{\sum }\tau h_{S}\left(x_{p}\left(t_{m}\right)-x_{k\ell }\right)\;+\underset{q\in \text{Ω}}{\sum }\beta h_{S}\left(x_{q}\left(t_{m}\right)-x_{k\ell }\right).$$

The echo signal in (4) describes a weighted coherent summation of SSF values at tissue and blood scatterer positions in Ω. The quantity |**x**_*n*_(*t_m_*) − **x**_*k*ℓ_| measures the distance between the *n*th scatterer and the pulse center located at (*x_ℓ_*, *z_k_*) during the time when the *m*th frame is recorded. With functional representation for *h_S_*, the echo signal for continuously varying scatterer positions is simulated.

The spatial distribution of scatterers modeled in this way is represented by an independent, multivariate Poisson variable. The acquisition noise is given by an independent, zero-mean, multivariate normal variable. Independence ensures that both frequency spectra are constant over the measurement bandwidth. Hence, the ensemble echo spectrum of this linear system is the SSF spectrum scaled by a flat scatterer-field spectrum, and that product is added to a band-limited white noise spectrum.

### C. Gabor Approximation to the SSF

The Gabor approximation to the SSF for a linear array transducer is

(5) $$h_{G}\left(x|u_{0},\sigma_{x},\sigma_{z},\phi \right)=\sin\left(2\pi u_{0}z+\phi \right)\times \frac{1}{{\left(2\pi \right)}^{3/2}\left(2\sigma_{x}^{2}\right)\sigma_{z}}\times \exp\left[-\frac{1}{2}\left({\left(\frac{x}{\sigma_{x}}\right)}^{2}+{\left(\frac{y}{2\sigma_{x}}\right)}^{2}+{\left(\frac{z}{\sigma_{z}^{2}}\right)}^{2}\right)\right]$$

where *u*_0_, *σ_x_*, *σ_z_*, and *φ* are free parameters. The approximation provides a relatively simple close-form expression to estimate the pulse parameters described in Section II-G.

We computed the SSF function *h_S_* from Rayleigh–Sommerfeld diffraction theory (see [27, eq. (9)]) to compare with the Gabor approximation from (5). The approximation *h_G_* was found by adjusting the four Gabor parameters to minimize the squared error

$${\left\|h_{S}\left(X_{\ell },z_{k}\right)-h_{G}\left(x_{\ell },0,z_{k}|u_{0},\sigma_{x},\sigma_{z},\phi \right)\right\|}^{2}.$$

**Fig. 1.**
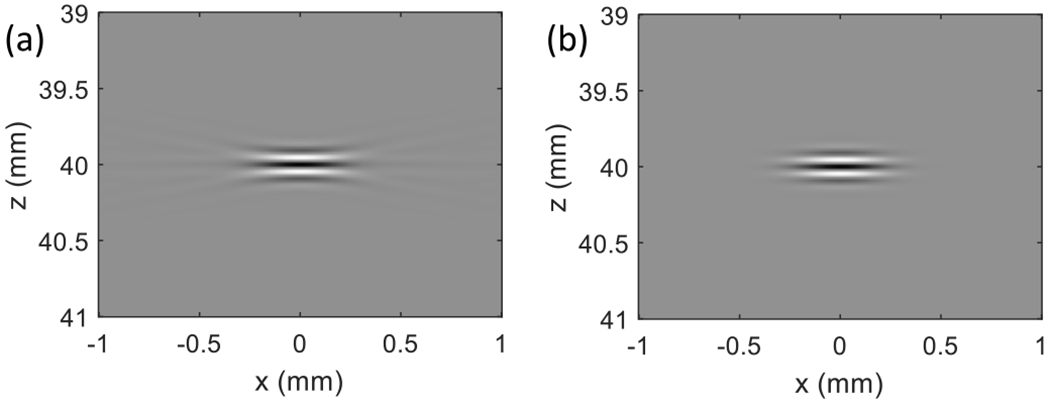
(a) 8-MHz SSF with 60% fractional bandwidth and f-number = 2 in-plane focusing and (b) its Gabor-function approximation.

Fig. 1(a) is an example of the SSF computed using the Rayleigh–Sommerfeld theory for a nominal 8 MHz, 60% fractional bandwidth pulse, and f-number = 2 in-plane aperture. The f-number is the ratio of focal length to active aperture length. We assumed the attenuation coefficient, *α* = *α*_0_*z*_0_*f*_0_, where *α*_0_ = 0.5 dB/cm-MHz, *z*_0_ = 4.0 cm focal length, and *f*_0_ = 8 MHz center frequency of the transducer. The frequency-dependent scattering amplitude factor was *f*^1.3^. The best-fit Gabor approximation, as shown in Fig. 1(b), has a center frequency of 7.32 MHz and a fractional bandwidth of 0.63. Given the similarity of pulses, we used *h_G_* in place of *h_S_* in (4) to simulate echo signals.

### D. Tissue and Blood Cell Motion

The model assumes that echo frames are recorded instantaneously as scatterers move continuously in Ω. The locations of tissue and blood scatterers in the *m*th echo frame are

(6) $$x_{p}\left(t_{m}\right)=\left(m-1\right)T_{m}v_{p}\left(t_{m}\right)+x_{p}\left(t_{1}\right)\;x_{q}\left(t_{m}\right)=\left(m-1\right)T_{m}\left(v_{p}\left(t_{m}\right)+v_{q}\left(t_{m}\right)\right)+x_{q}\left(t_{1}\right).$$

The velocity vector for tissue scatterers **v**_*p*_(*t_m_*) [mm/s] varies in time, but all tissue scatterers move together (rigid translation). In contrast, the time-varying velocity of blood scatterers **v**_*q*_(*t_m_*) allows each blood cell to move independently in space. Because they perfuse the moving tissues, blood cell motion is determined by the sum of both velocities.

The simulations include two patterns of tissue (clutter) motion, both have all scatterers moving rigidly between frames, i.e., **v**_*p*_(*t_m_*) = **v**_*p*′_(*t_m_*) ∀ {*p, p*′} ∈ Ω. In some cases, the transducer probe is *translated linearly* along one of three axes. In other cases, scatterers *oscillate rigidly* in the scan plane [see Fig. 5(b)] to mimic breathing and cardiac pulses.

Two blood-cell velocity patterns were modeled. First, *directed flow* data simulated all blood cells moving along the beam axis at the same speed but each with a ±15° random angular variability about the axis, e.g., Fig. 6(a). Second, *diffuse flow* data simulated blood cells moving in uniformly random directions in the volume at constant speed, e.g., Fig. 7(a). The spatially averaged blood velocity in diffuse flow is zero.

### E. Displacement Estimation and Spatial Registration

From (4), echo signals within the first and *m*th frames are

(7) $$g_{k\ell 1}=\epsilon_{k\ell 1}+\overset{N}{\underset{n=1}{\sum }}a_{n}\;h\left(x_{n1}-x_{k\ell }\right)\;g_{k\ell m}=\epsilon_{k\ell m}+\overset{N}{\underset{n=1}{\sum }}a_{n}\;h\left(x_{n1}+\text{Δ}x_{1m}-x_{k\ell }\right)$$

where **x**_*n*1_ ≜ **x**_*n*_(*t*_1_). From (6), the 3-D translation of the *m*th frame relative to the first frame is

$$\text{Δ}x_{1m}=x_{p}\left(t_{m}\right)-x_{p}\left(t_{1}\right)=\left(m-1\right)T_{m}v_{p}\left(t_{m}\right).$$

Only the velocity of the more echogenic clutter scatterers is considered for the purpose of registration.

Tissue scatterer displacement Δ**x**_1*m*_ is estimated in two stages. First, we compute the average discrete 2-D spatial cross correlation function *φ* between regions in the first and *m*th RF echo frames. If *k* and *ℓ* are the axial and lateral spatial coordinate indices for the first frame, *k*′ and *ℓ*′ are the indices for the *m*th frame, and Δ*k* = *k* − *k*′ and Δ*ℓ* = *ℓ* − *ℓ*′ are the differences. For jointly wide-sense stationary echo data *g* in analysis area *X*_0_ × *Z*_0_

(8) $$\phi_{\text{Δ}k\text{Δ}\ell 1\;m}={𝓕}^{-1}\left\{𝓕\left\{g_{k\ell 1}\right\}{\left(𝓕\left\{g_{k\prime \ell \prime m\prime }\right\}\right)}^{*}\right\}.$$

*𝓕*{·} denotes the forward 2-D *discrete Fourier transform* of zero-padded echo data for the region, *𝓕*^−1^{·} is the inverse transform, and * indicates the complex conjugate. The *coarse displacement estimate* is indicated by the sample indices at the correlation peak using

(9) $${\left[\text{Δ}\hat{k},\text{Δ}\hat{\ell }\right]}_{1m}=\arg\;\underset{\text{Δ}k,\text{Δ}\ell }{\max}\;\phi_{\text{Δ}k\text{Δ}\ell 1m}.$$

Equation (9) estimates the in-plane translation of regions between frames 1 and *m* in units of discrete lateral and axial RF echo samples. For example, $\text{Δ}\hat{k}=\text{Δ}\hat{\ell }=0$. For |**v**_*p*_| > 0, discrete displacements are estimated with in-plane spatial resolution given by the RF echo sampling intervals, (*X, Z* = *cT_k_*/2).

Second, the *subsample displacement estimate* is found by combining a cubic spline interpolation algorithm with a 2-D unconstrained nonlinear optimization search algorithm implemented in the MATLAB function fminsearch. At each step in the search, the *m*th echo frame is continuously interpolated via MATLAB’s interp2 before applying a derivative-free simplex search method [33]. The fine-scale search begins at the coarse-scale estimate values and terminates once the convergence criterion of 10^−4^ is obtained. The result is the subsample lateral and axial displacement estimates ${\left(\hat{\xi }\prime ,\hat{\zeta }\prime \right)}^{\text{⊺}}$ respectively.

The *continuous displacement estimate* [mm] between frames 1 and *m* is the sum of the coarse and fine estimates

(10) $${\hat{\xi }}_{1m}=\left(\begin{array}{c} {\hat{\xi }}_{1m}\\ {\hat{\zeta }}_{1m} \end{array}\right)=\left(\begin{array}{c} \text{Δ}\hat{\ell }X+\hat{\xi }\prime \\ \text{Δ}\hat{k}\frac{cT_{k}}{2}+\hat{\zeta }\prime \end{array}\right).$$

Tissue scatterers can move in three spatial dimensions, but only displacements in the scan plane can be measured with a 1-D array transducer. Displacement estimation is successful if ${\hat{\xi }}_{1m}≃\text{Δ}x_{1m}$.

**Fig. 2.**
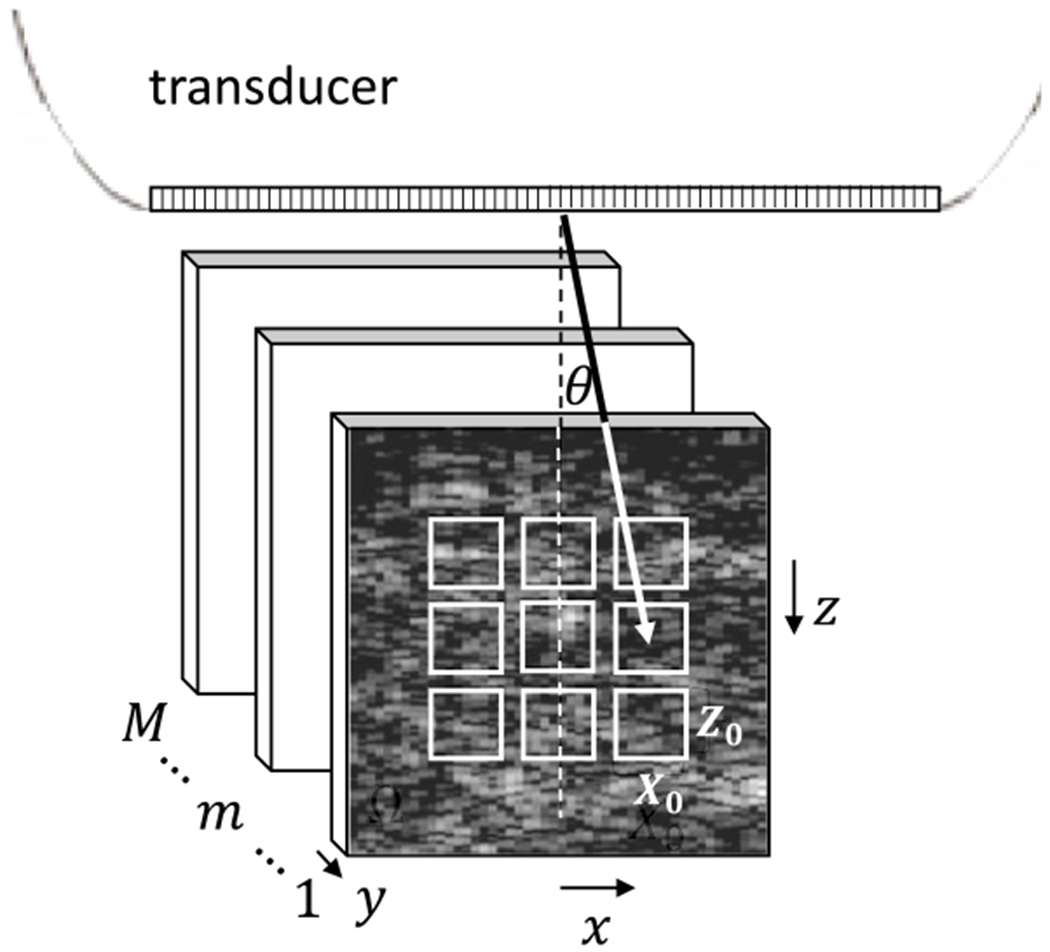
Geometry of simulated echo acquisition in the (*x, z*) scan plane. Displacements are estimated in nine distinct regions of area *X*_0_ × *Z*_0_ for each frame in the *M*-frame ensemble relative to the first.

The simulated scattering object is 14 mm × 14 mm × 5 mm, where the larger two dimensions form the *x, z* scan plane. The resulting echo frame from (4) is 10 mm × 10 mm. The displacement of the frame used for spatial registration is the average value estimated from nine nonoverlapping regions in each frames, each of area *X*_0_ × *Z*_0_ (nominally 1 mm^2^), as illustrated in Figs. 2, 6(a), and 7(a). Because simulated tissue echoes are wide-sense stationary processes, the exact placement of the subregions is arbitrary. Region selection needs more carefully consideration for in vivo measurements where tissue echogenicity is heterogeneous. Each frame in the ensemble is aligned with the first frame by removing the relative displacement via interpolation methods.

### F. Estimator Efficiency

An efficient unbiased displacement estimator for registering echo frames exhibits a measurement variance that approaches the lower statistical bound on variance for the parameters of the experiment. Evaluating estimator efficiency is also a way to validate the echo simulation and displacement estimation algorithms. Knapp and Carter [35] derived the Cramér–Rao lower bound (CRLB) on the variance of correlation-based time delay estimates for a linear signal model. This section explains how (4) may be adapted to their CRLB variance expression in the spatial frequency domain.

Let **u** = (*u_x_*, *u_z_*)^⊺^ be the continuous spatial-frequency vector for the *x, z* plane. The 2-D *discrete-time Fourier transform* of sampled RF echo data from the *m*th frame is ${\hat{G}}_{m}\left(u\right)=𝓕\left\{g_{k\ell m}\right\}$. Here, 2-D echo samples in space are transformed into the continuous 2-D spatial frequency domain. The auto power-spectral density for the *m*th frame is $P_{mm}\left(u\right)=\varepsilon \left\{{\left|{\hat{G}}_{m}\left(u\right)\right|}^{2}\right\}$, and the cross power-spectral density between the first and *m*th frames is $P_{1m}\left(u\right)=\varepsilon \left\{{\hat{G}}_{1}\left(u\right){\hat{G}}_{m}^{*}\left(u\right)\right\}$. *ε*{⋅} is the expectation operator. Displacements are assumed to be small compared to the correlated region size.

The cross correlation between echo data in frames 1 and *m*, now expressed using continuous position variables, is related to the cross-spectral density via the 2-D integral transform

$$\phi_{1m}(\xi )=\int_{-\infty }^{\infty }du\;P_{1m}(u)e^{i2\pi u^{⊤}\xi }.$$

The *magnitude-squared coherence function* between data in frames 1 and *m* is *C*_1*m*_. It is a function of the cross-spectral and autospectral densities [35]

(11) $$C_{1m}(u)=\frac{{\left|P_{1m}(u)\right|}^{2}}{P_{11}(u)P_{mm}(u)},\;0\leq C_{1m}(u)\leq 1.$$

*C*_1*m*_ measures the extent to which data in the *m*th echo frame can be predicted from data in the first frame: *C*_1*m*_ = 0 at all spatial frequencies for statistically independent data and *C*_1*m*_ = 1 for identical data, e.g., *m* = 1.

The CRLB on variance for in-plane displacements estimated along the *x*- and *z*-axes is, respectively, [35]

(12) $$\mathrm{var}(\hat{\xi })\geq -ℰ\left\{{\left|\frac{\partial^{2}}{\partial \xi^{2}}\ln\;p\left(G|\sigma_{f},\sigma_{\epsilon },\xi \right)\right|}^{-1}\right\}\;={\left[8\pi^{2}X_{0}\int_{0}^{\infty }du_{x}\;u_{x}^{2}\frac{C_{1m}\left(u_{x}\right)}{1-C_{1m}\left(u_{x}\right)}\right]}^{-1}\;\mathrm{var}(\hat{\zeta })\geq {\left[8\pi^{2}Z_{0}\int_{0}^{\infty }du_{z}\;u_{z}^{2}\frac{C_{1m}\left(u_{z}\right)}{1-C_{1m}\left(u_{z}\right)}\right]}^{-1}$$

where **G** = (*G*_1_(*u_x_*), *G_m_*(*u_x_*))^⊤^ and *p*(**G**|*σ_f_, σ_ϵ_, ξ*) is a conditional normal probability density for the data—the likelihood function. The integral exists when the signal includes bandlimited white noise because *C*_1*m*_ ≠ 1. The ensemble spectral properties of simulated echo data are known.

For the linear signal model and wide-sense stationary multivariate processes of (4), autospectral densities have signal and noise terms given by

(13) $$P_{11}(u)=P_{mm}(u)=P_{h_{mm}}(u)+P_{\epsilon_{mm}}(u)=\sigma_{f}^{2}|H(u){|}^{2}+\frac{\sigma_{\epsilon }^{2}}{B_{x}B_{z}}.$$

Applying (5), we have $H(u)≜H\left(u_{x},0,u_{z}\right)=𝓕\left\{h_{G}(x)\right\}$. *B_x_* and *B_z_* [mm^−1^] are effective noise bandwidths along the *u_x_*- and *u_z_*-axes. The effective noise bandwidth is traditionally defined for positive frequencies as [34]

(14) $$B≜\frac{1}{|H(0){|}^{2}}\int_{0}^{\infty }du|H(u){|}^{2}.$$

For bandpass white noise, bandwidths are determined by the sampling rates (see the values listed in Table I).

The cross-spectral density for the two frames is

(15) $$P_{1m}(u)=P_{11}(u)e^{-i2\pi u^{⊤}\text{Δ}x_{m}}+P_{\epsilon_{1m}}(u)\;=\sigma_{f}^{2}|H(u){|}^{2}e^{-i2\pi \left(u^{⊤}\text{Δ}x_{m}\right)}$$

because, for uncorrelated noise, *P*_*ϵ*_1*m*__(**u**) = **0**.

Combining (11) with (13)–(15), we find

(16) $$C_{1m}\left(u_{x}\right)=\frac{S\left(u_{x}\right)}{1+S\left(u_{x}\right)}\;\text{and}\;C_{1m}\left(u_{z}\right)=\frac{S\left(u_{z}\right)}{1+S\left(u_{z}\right)}$$

where

(17) $$S\left(u_{x}\right)=B_{x}\frac{\sigma_{f}^{2}}{\sigma_{\epsilon }^{2}}\int_{0}^{\infty }du_{z}|H(u){|}^{2}\;S\left(u_{z}\right)=B_{z}\frac{\sigma_{f}^{2}}{\sigma_{\epsilon }^{2}}\int_{0}^{\infty }du_{x}|H(u){|}^{2}.$$

*S*(**u**) is the ratio of the signal spectrum to the noise spectrum. Separately integrating the numerator and denominator of *S*(**u**) over positive frequencies results in the echo SNR

(18) $$\text{SNR}=\frac{\sigma_{f}^{2}\int_{0}^{\infty }du|H(u){|}^{2}}{\frac{\sigma_{\epsilon }^{2}}{B_{x}B_{z}}\int_{0}^{\infty }du}=\frac{\sigma_{f}^{2}}{\sigma_{\epsilon }^{2}}\int_{0}^{\infty }du|H(u){|}^{2}.$$

Finally, combining (12) with (16) and (17) gives the lateral and axial displacement variance bounds [mm^2^], respectively,

(19) $$\mathrm{var}(\hat{\zeta })\geq {\left[8\pi^{2}X_{0}\int_{0}^{\infty }du_{x}\;u_{x}^{2}S\left(u_{x}\right)\right]}^{-1}\;\mathrm{var}(\hat{\zeta })\geq {\left[8\pi^{2}Z_{0}\int_{0}^{\infty }du_{z}\;u_{z}^{2}S\left(u_{z}\right)\right]}^{-1}.$$

Equation (19) shows that unbiased displacement errors are functions of the correlation area, pulse properties, sampling rates, and echo SNR. It also shows that the variance bound is independent of the true displacement Δ**x**_*m*_.

### G. Echo-Signal Model Parameters

The Gabor pulse approximation of (5) makes it convenient to estimate closed-form expressions for the terms in (19) that defines simulated signal properties. The independent parameters listed in Table I were selected to represent realistic spatial sampling and pulse parameters for 5- and 20-MHz broadband (bb) pulses. From these values, we estimate the lateral *σ_x_*, elevational *σ_y_*, and axial *σ_z_* Gabor pulse parameters, noise bandwidths *B_x_*, *B_z_*, the 2-D speckle correlation area *A*_2-D_ and 3-D speckle correlation volume *A*_3-D_. We assume that the lateral beamwidth is specified by its full-width-at-half-maximum (FWHM) value, FWHM_*x*_ = *cz*_0_/*f*_0_*D_x_* = 2 × f-number/*u*_0_, where *z*_0_ is the focal length and *D_x_* is the lateral active-aperture length. The pulse bandwidth is determined in the spatial frequency domain by FWHM*_u_z__* of the axial pulse spectrum |*H*(*u_z_*)|^2^. Noise variance $\sigma_{\in }^{2}$ is computed from the echo SNR equation of (18) after computing the average signal power $\sigma_{f}^{2}\int du{\left|H\left(u\right)\right|}^{2}$ from the simulated noise-free echo signals of (4). Selecting at least 20 scatterers per pulse, the total number of scatterers in the simulated volume is found from expressions for the speckle correlation volume *A*_3-D_, as listed in Table I. *A*_3-D_ includes 95% of the 3-D Gabor pulse energy.

The fractional bandwidth for bb 5-MHz pulses was set to 0.75 for an echo SNR estimated experimentally at 30 dB. The bb pulses provide more precise spatial registration results at high SNR. The fractional bandwidth of 20-MHz pulses was lowered to 0.4 for an echo SNR estimate of 15 dB.

### H. Phantom Measurements

A gelatin phantom containing a spatially fixed random suspension of cornstarch particles as tissue scatterers (no blood-mimicking scatterers) was scanned to experimentally verify the simulation results for clutter displacement estimation. The construction of the gelatin materials follows methods described previously [29]. We added 2.6% cornstarch by mass to the 125-cm^3^ phantom volume. The density of dry cornstarch is 0.625 g/cm^3^. Particle sizes are modeled as a Gaussian distribution of spheres with the mean diameter of 18 *μ*m, the standard deviation of 4 *μ*m, and the range of 2–30 *μ*m [36] so that the particle density is on the order of 10^4^ per mm^3^ of gel. The speed of sound in the phantom is 1506 ± 1 m/s, and the slope of the frequency-dependent attenuation coefficient is 0.30 ± 0.03 dB cm^−1^ MHz^−1^ [32].

The 5-cm gelatin cubes were scanned with a Sonix RP ultrasonic imaging system (Ultrasonix Medical Corporation, Richmond, BC, Canada) at 5 MHz and a depth of 4 cm. The scan plane area over which displacement was estimated from the RF echo signals was *X*_0_ = 12.52 mm × *Z*_0_ = 9.70 mm with lateral and axial sampling intervals *X* = 0.3438 mm and *Z* = 0.0419 mm. A linear array transducer attached to a three-axis motion controller unit was oriented such that the beam axis was perpendicular to an acrylic plate on which the phantom rested. In this fixture, RF echo data were recorded in color mode as the transducer was translated along the beam (*z*-axis), perpendicular to the beam in the scan plane (*x*-axis), or perpendicular to the beam and normal to the scan plane (*y*-axis). The phantom contained a weakly reflecting cylinder with a long axis oriented collinear to the *x*-axis of the transducer to help lateral translations remain in the scan plane.

As the transducer was moved along the *z*-axis at 0.48 mm/s, the scanner recorded 12 frames/s, yielding 25 frames/mm. Alternatively, the transducer translated along the *x*- or *y*-axes at 1.0 mm/s, while the scanner recorded ten frames/s, yielding ten frames/mm. We selected echo frames during times of constant transducer velocity; the results for axial and lateral axis motion are summarized in Fig. 4.

The interframe correlation coefficient between the first and *m*th frames, *ρ*, was computed as the transducer moved along each axis to estimate the pulse dimensions; for example, see *ρ*(*y*) in (Fig. 10(b) and (d). The correlation coefficient functions were each fit to a Gaussian, and the FWHM value was found. Applying the Gaussian-function relation $\text{FWHM}=\text{2}\sqrt{2\;\ln\;2}\times \sigma$, we estimate the following color-mode pulse parameters for the experiment: *σ_x_* = 0.251 mm, *σ_y_* = 0.743 mm, and *σ_z_* = 0.163 mm. The experimental parameters for narrowband (nb) color-mode acquisitions may be compared to the bb 5-MHz simulation values listed in Table I.

## III. Results

### A. Uncertainties in Registering Clutter Echoes at 5 MHz

**Fig. 3.**
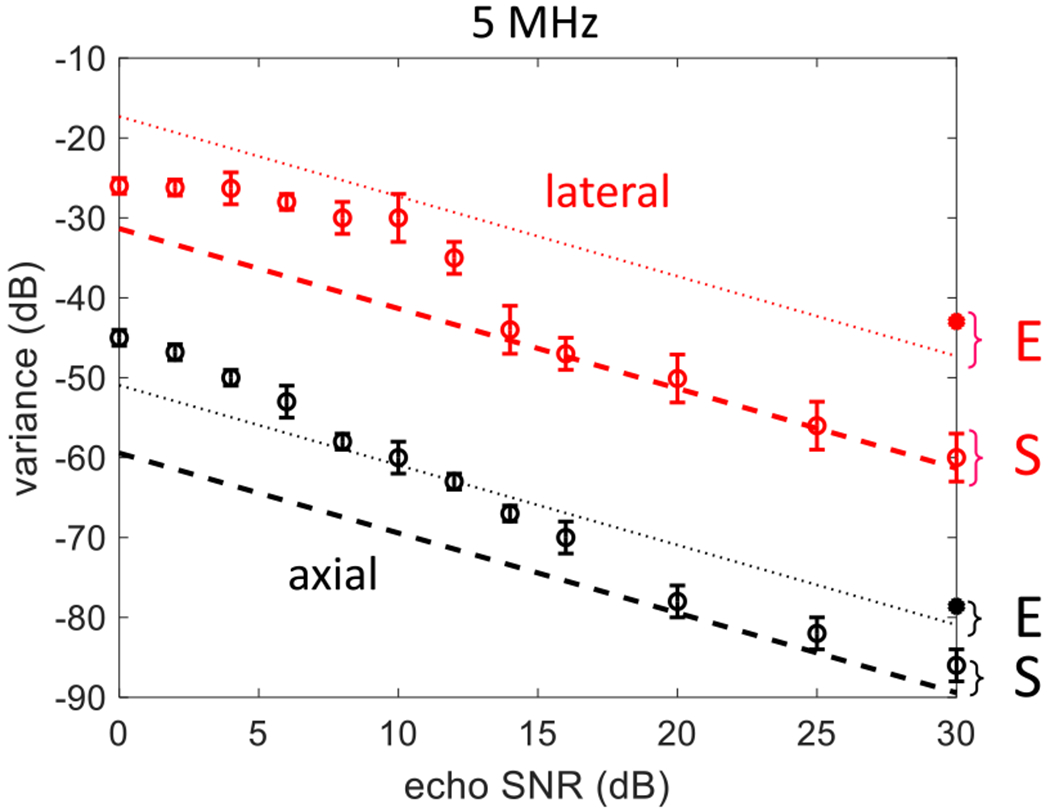
In-plane displacement variances are estimated from simulated and experimental data at 5 MHz. Variances from echo simulations are estimated for lateral (open red circles) and axial (open black circles) motions, where the transducer was moved along one axis as the scatteringmedium remained fixed. The two solid circular points at SNR = 30 dB are variances estimated experimentally. The dashed lines are the CRLBs for the simulation parameters (S), the dotted lines are for experimental (E) parameters, and both are computed using (19).

Fig. 3 displays the CRLB on variance from (19) for lateral displacements estimates (red lines) and axial displacement estimates (black lines) over an echo SNR range of 0–30 dB. The dashed lines are for simulated echo signals (S) computed from the 5-MHz parameters listed in Table I, while the dotted lines are for 5-MHz experimental echo signals (E) computed from the parameters described in Section II-H. For a fixed echo-signal strength, the variance bounds are proportional to the acquisition noise variance *σ_ϵ_*^2^, giving the linear decrease with increasing SNR viewed on a log-log scale. The quadratic frequency weighting in the variance expressions of (19) means that the axial variances are about 30 dB lower than lateral variances.

On the same plot are displacement variances measured from simulated echo data over the SNR range (open circle points) and experimental echo data at SNR ≃ 30 dB^2^ (closed circle points). Variances measured using 5-MHz echo simulations approach the lower bound when the echo SNR exceeds 20 dB. At lower SNR values, ambiguity errors [30] increase the variance.

The simulation results in Fig. 3 show that the displacement estimator from (10) is efficient for data simulated by the echo model of (4). Achieving the lower bound for echo simulations is a consistency check of the modeling and estimation programming. It shows that spatially coherent in-plane object displacements are reversible by spatially registering echo signals, within the noise limitations responsible for the estimation variance. When diffuse blood perfusion or other spatially incoherent scatterer motion is present, they too increase the displacement variance for spatial registration of clutter echoes.

The experimental results in Fig. 3 show that displacement variances (two solid points at SNR = 30 dB) are greater than those estimated from simulated echo signals. Similarly, the variance bounds using experimental parameters (dotted lines) are larger than those obtained with simulation parameters (dashed lines). The increases are mostly explained by the following differences between experimental and simulated echo data parameters. First, the color-mode pulses of the experiment extend in space more than the simulated B-mode pulses. The nb color-mode RF echo signals generate more displacement uncertainty than the bb signal at high SNR, but they enable spatial registration and PD-US estimation in one dataset. Second, the area over which echo signals are spatially averaged and the sampling intervals differ; the latter influences noise power calculations for (19). Third, the experimental pulse spectrum must include the influence of ultrasonic attenuation that modulates the spectral amplitude of the pulse along the *u_z_*-axis. We did not model attenuation losses in the simulated echo data. The differences between the variance bounds and the variance estimates for phantom data are 2 dB axially and 6 dB laterally.

**Fig. 4.**
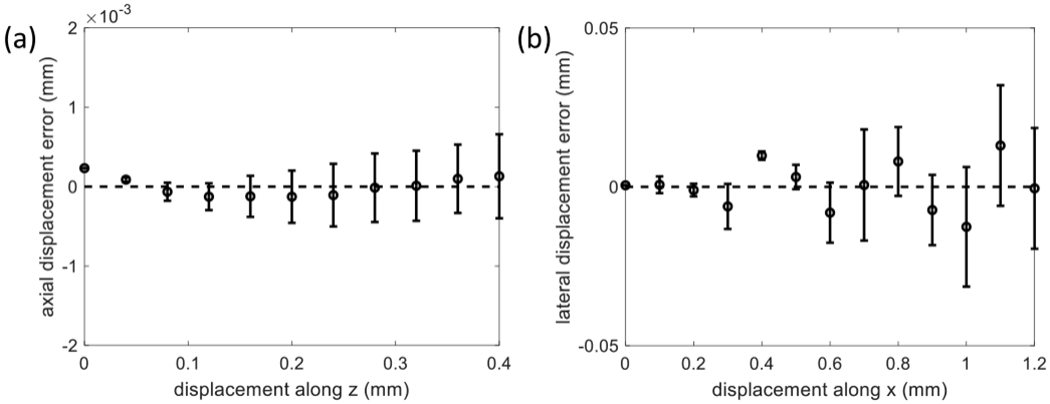
(a) Axial and (b) lateral displacement errors (±1 sd) are shown for phantom experiments at 5 MHz where the echo SNR is 30 dB. Clutter motion is a linear translation along the *z*- or *x*-axis as indicated. Measurement variances reported as solid points in Fig. 3 are computed from these errors.

**Fig. 5.**
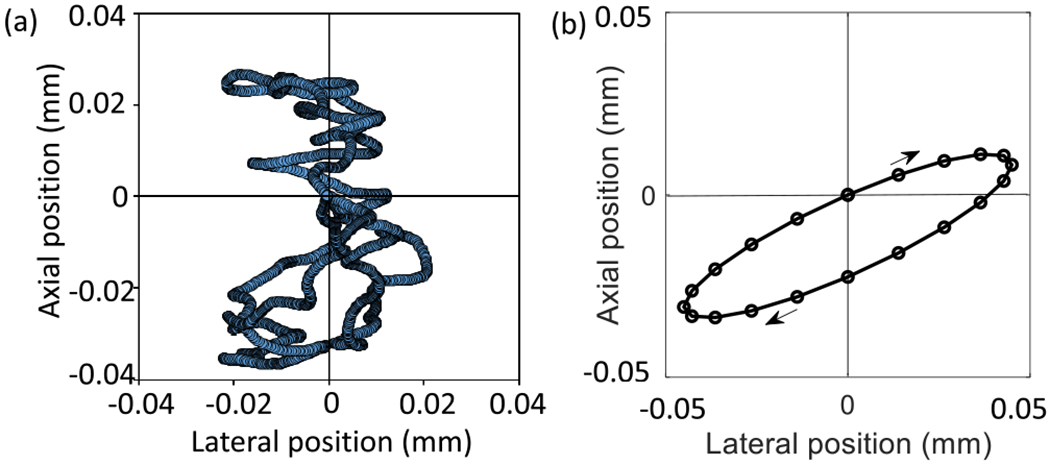
(a) In vivo measurements of clutter motion are made at 8 MHz in a transverse scan plane of the human calf muscle. In the experiment, the subject’s leg and the probe are both fixed. (b) Oscillating in-plane clutter motion applied in echo simulations is shown. The oscillation frequency is 1.0 Hz.

Fig. 4 shows examples of registration errors resulting from the phantom experiments. The translation ranges are limited because clutter alignment in vivo typically involves translations much less than 1 mm [see Fig. 5(a)]. The lateral error plotted in Fig. 4(b), for example, is $e_{x}={\hat{\xi }}_{1m}-\text{Δ}x_{1m}$. We linearly detrend *e*_*x*_ before estimating the lateral displacement variance $\mathrm{var}(\hat{\xi })=\left(1/N_{p}\right)\sum_{j=1}^{N_{p}}e_{x}^{2}$ reported in Fig. 3. *N*_*p*_ is the number of measurements along the displacement axis. Estimates of $\mathrm{var}\left(\hat{\xi }\right)$ in Fig. 4(a) are found similarly. The error bars shown in Fig. 4 indicate that displacement errors become more variable as the translational distance increases, a situation not predicted by the CRLB equations. Our explanation for the increasing error is addressed in Section III-G.

### B. In-Plane Clutter Motion Patterns

Clutter motion patterns of two types were observed during in vivo scanning. Motion is smallest when a prone subject has their leg gently restrained, and the probe is rigidly fixed after being acoustically coupled to a relaxed calf muscle. Under these conditions, muscle movements relative to the beam axis follow a small-range chaotic pattern like the example of Fig. 5(a). For echo simulations, the oscillating in-plane tissue motion pattern shown in Fig. 5(b) was selected with an oscillation frequency of 1.0 Hz.

### C. 5-MHz Echo Simulations With Directed Perfusion

**Fig. 6.**
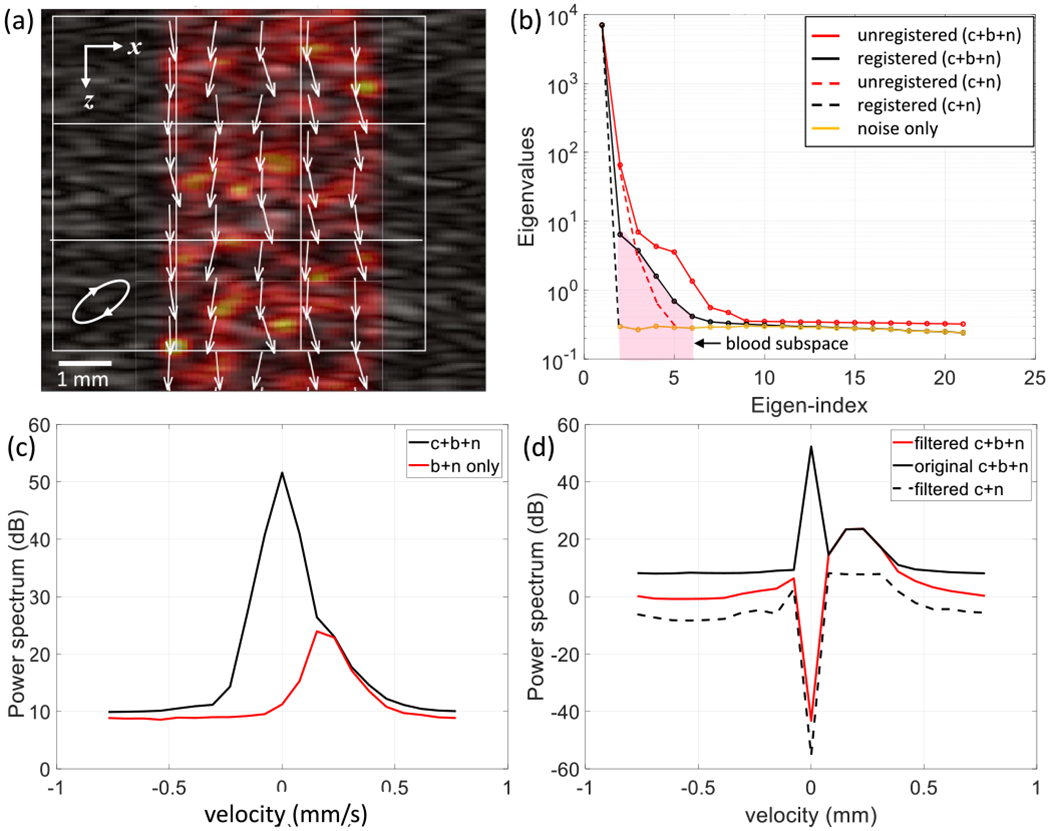
Simulated PD-US image and spectra for directed perfusion with the blood-cell speed of 0.2 mm/s at 5 MHz and 30-dB SNR. The echo signals for the image in (a) were spatially registered and PCA filtered. The tissue scatterers (gray scale) include a central region with simulated blood perfusion (color overlay). The motion patterns for clutter and blood are indicated by white arrows along with the nine regions where displacement is estimated for spatial registration. (b) Eigenspectra are shown from the PCA filter for unregistered and registered echo signals that include clutter, blood, and noise (c + b + n) sources, clutter and noise (c + n, no blood component), and noise only. PCA filter thresholds at eigenindices 2 and 6 clearly define the blood subspace for directed perfusion once the echo frames are registered. (c) Doppler frequency spectra are shown for clutter, blood, and noise sources (c + b + n) and for blood and noise sources (b + n) before spatial registration and filtering. (d) Doppler frequency spectra are shown for spatially registered echo signals. The solid black and red lines are the spectra of echo signals with clutter, blood, and noise sources before and after PCA filtering, respectively. The dashed black line shows the registered and filtered spectrum when the echo signal contains only clutter and noise. The Doppler frequency axis was converted into scatterer velocity using *v* [mm/s] = (*c*/2) × (Doppler frequency [Hz]/pulse frequency [MHz]).

Fig. 6 summarizes PD-US results for 5-MHz echo simulations, where a clutter field includes a central region of *directed perfusion*. The oscillating clutter motion between frames in the simulated echo ensemble is spatially registered to the first frame based on the average displacement detected in nine 2 × 2 mm windows placed inside and outside the region of perfusion [see Fig. 6(a)]. We divide the total region into nine windows and average to also estimate displacement variance. The speed of each blood cell is 0.2 mm/s, directed on average downward with a ±15° variation about the *z*-axis. Scatterers are translated in the scan plane in an elliptical pattern to simulate cyclic clutter motion. The simulated 21-frames ensemble was acquired over 2 s.

In Fig. 6(b), the eigenspectrum of the combined clutter, blood, and noise echo signal (c + b + n) is examined with and without spatial registration. In the same plot, the eigenspectrum of echo signals composed of only clutter and noise (c + n) before and after registration are compared along with a noise-only spectrum. These plots show that spatial registration narrows the echo eigenspectra when there is coherent motion, concentrating clutter energy primarily in the first eigenvalue. Following registration, the subspace dominated by directed blood perfusion is contained within eigenvalues 2–6. Eigenvalues outside this range are set to zero when forming the image of Fig. 6(a).

Fig. 6(c) and (d) shows the Doppler frequency spectra for the same echo signals. We see that clutter and blood components that are broadened by clutter motion [see Fig. 6(c)] are narrowed and separated by spatial registration [see Fig. 6(d)] without distorting the mean blood-cell speed. The blood component of the spectrum also narrows when the blood motion coupled to tissue motion is eliminated. Clutter filtering of the spatially registered Doppler spectrum seen in Fig. 6(d) is effective at separating the clutter and directed-perfusion components. When the blood component is excluded (filtered c + n), the clutter filter passes noise in the velocity range of blood-cell motion.

### D. 5-MHz Echo Simulations With Diffuse Perfusion

**Fig. 7.**
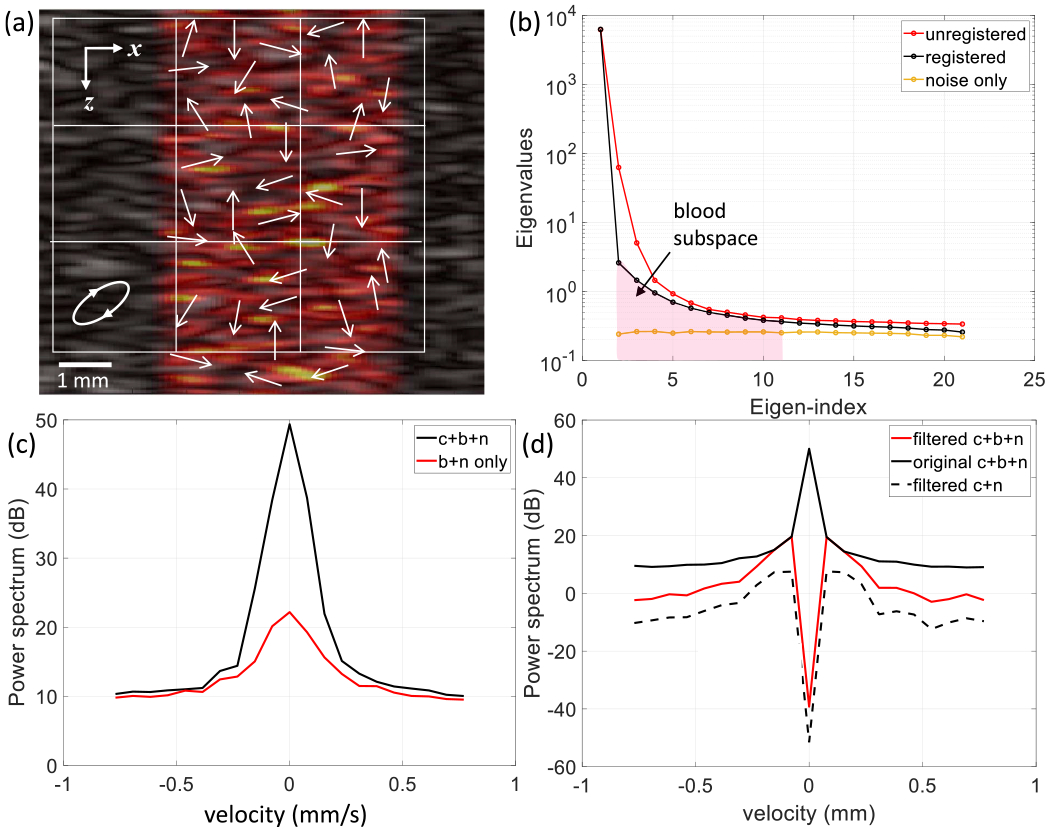
Simulated PD-US image and spectra for diffuse perfusion with the blood-cell speed of 0.2mm/s at 5MHz and 30-dB SNR. The PCA filter thresholds are expanded to 2–11. Other parameters and each subsection are the same as those described in Fig. 6.

Analogous to the results in Fig. 6, Fig. 7 summarizes PD-US results for 5-MHz echo simulations, where the clutter field contains a central region of *diffuse perfusion*. As with directed perfusion, the frames are spatially registered before clutter filtering that eliminates the first eigenvalue.

The interface between the blood and noise subspaces in the diffuse-perfusion eigenspectrum of Fig. 7(b) is less distinct than for directed perfusion. The greater diversity of the blood cell movements in diffuse perfusion expands the blood subspace, and so we adjusted the eigenindices on the clutter filter to the range of 2–11. This choice was a compromise between losing too much blood power and including too much noise power.

The clutter and blood components of the Doppler frequency spectrum [see Fig. 7(c)], which overlapped entirely before spatial registration, are more distinct after spatial registration [see Fig. 7(d)]. It was unclear to us how much of the registered and filtered spectrum [solid red line in Fig. 7(d)] is from the blood component. Thus, we removed the blood component and reprocessed the clutter in noise signals [dashed black line in Fig. 7(d)]. The difference between the solid red line and dashed black line spectra estimates the blood spectrum. It is important to note that eliminating blood scatterers changes the eigenvectors in the PCA filter, so comparisons are not exact. Nevertheless, we see how the noise power is enhanced in the frequency channels of the blood spectrum, as occurred with directed perfusion.

Our observations of Doppler spectra from in vivo peripheral perfusion data [17], [18] made before and after spatial registration are generally symmetric about the origin, suggesting that in vivo muscle perfusion is diffuse. Spectral symmetry is the reason for power-Doppler methods being more informative than color-flow methods in perfusion imaging applications. Similar to the simulated spectra in Fig. 7(d), spatial registration narrows the clutter components. However, these effects are difficult to quantify in vivo because the component contributions are unknown.

### E. Echo Signal Power at 5 MHz

**Fig. 8.**
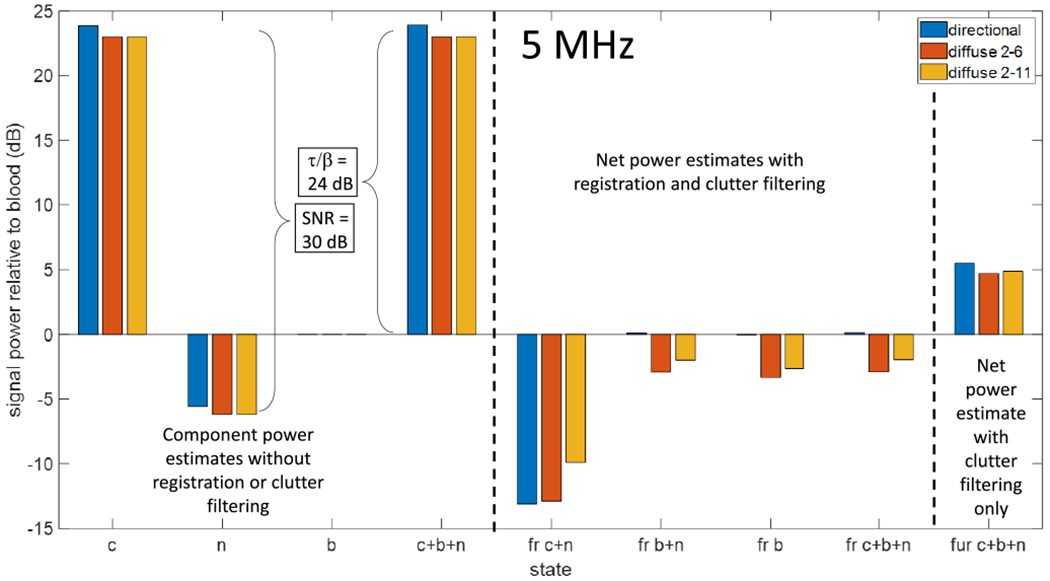
Measurements of net signal power for the 5-MHz, 30-dB SNR echo simulations of Figs. 6 and 7. As listed in Table I, the ratio of tissue to blood reflectivities *τ/β* = 24 dB. Nine experimental states are examined, where, in each case, signal power is estimated relative to the bloodonly signal power. The first four states are the unregistered, unfiltered echo data for (left to right) in-plane moving clutter (c), acquisition noise (n), and blood perfusion (b) as recorded separately and in combination (c + b + n). The next four states are the filtered and registered echo-signal power relative to blood power. The signal sources are clutter in noise (fr c + n), blood in noise (fr b + n), blood only (fr b), and all components in combination (fr c + b + n). The objective in these four states is to measure blood power, which is accurately estimated when the power value is 0 dB. The unregistered, PCA filtered power from the combined signal is (fur c + n + b). Results for directed (blue) and diffuse (red and gold) perfusion are shown. The PCA filter passes eigenvalues of 2–6 (out of 21) for directed perfusion and 2–6 or 2–11 for diffuse perfusion.

Figs. 6 and 7 illustrate the effects of PCA filtering following spatial registration on Doppler frequency and eigenspectra for diffuse and directed perfusion in moving clutter and noise. However, the most important measure of filtering success for PD-US imaging is the net signal power relative to the true blood-signal power. Net signal power is the integral of the Doppler power spectrum over the measurement bandwidth. A strength of echo simulations is the ability to study the effects of spatial registration and PCA filtering on each component of the echo signal and various combinations. Fig. 8 displays the net signal power relative to blood power at 5 MHz for nine simulated measurement states with different combinations of signal components.

The signal power measured depends on whether the frames are spatially registered, the PCA filter thresholds, and the sources contributing to the echo signal. We report results for the two filter settings (2–6 and 2–11 for a 21-frame ensemble) that we investigated for directed and diffuse perfusion when comparing measurements with and without spatial registration. These comparisons quantify the effects of spatial registration and show the sensitivity of power estimates to the upper filter threshold.

The first four states on the left-hand side of Fig. 8 list the signal power from unregistered and unfiltered signals relative to the blood-echo power in decibels. The states examined are moving clutter (c), acquisition noise (n), and perfusing blood (b) components of the echo signal measured individually and in combination, labeled (c + b + n). Bar colors indicate measurements for directed and diffuse perfusion. Because these signals are unfiltered, the results for the two PCA clutter filter thresholds (red and gold bars) have the same value.

The next four measurement states in Fig. 8 display signal power after registration and filtering for clutter in noise (fr c + n), blood in noise (fr b + n), blood only (fr b), and for all three components (fr c + b + n). Note that the clutter filter eigenvectors change depending on which components are included in the echo signal, and yet, the expectation is that the blood-signal power is measured by each filtered signal that contains a blood component. Accurate blood-power measurements are those near 0 dB.

PCA filtering is effective for directed perfusion (blue bars) in any combination of echo components, provided that the blood subspace is made distinct by spatial registration of the frames. PCA filtering is less effective for diffuse perfusion. The filter with the narrower blood subspace (red bars) underestimates blood power by an average of 3 dB, while the broader subspace (gold bars) underestimates blood power by an average of 2 dB. If we increased the upper PCA-filter threshold, we would approach the blood power more closely. This is possible to do with simulation because we know the ground truth. In vivo, the echo SNR varies with signal strength and can be depth-dependent, which suggests that the upper filter threshold should be adjusted according to the circumstances. Our point is to demonstrate how blood and clutter scatterer movements couple with PCA filter thresholds to determine the PD-US signal.

### F. Echo Signal Power at 20 MHz

**Fig. 9.**
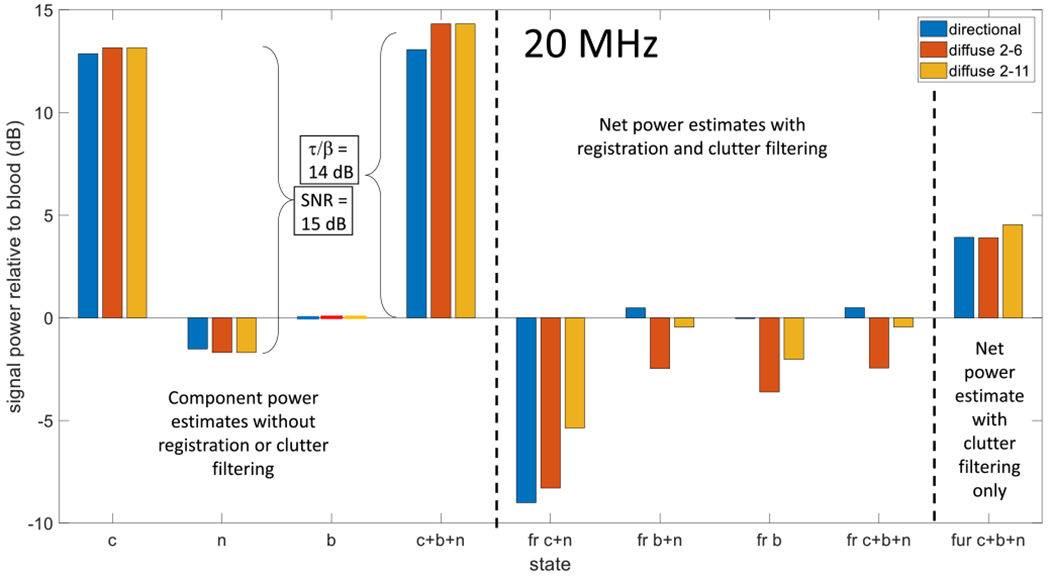
Measurements of net signal power for 20-MHz, 15-dB SNR echo simulations. The features of the bar graph are explained in Fig. 8.

PD-US methods are being developed using mouse models at higher pulse frequencies [17], [31]. For that reason, we repeated the 5-MHz results of Fig. 8 at 20 MHz and 15-dB echo SNR. Those findings are summarized in Fig. 9. We see that the echo SNR and *τ/β* ratio input into the simulator as a result of the change in pulse frequency are reflected in the output power measurements. We selected the same PCA filter thresholds since they are primarily determined by the movement patterns of source scatterers. Otherwise, the general trends observed at 5 MHz are seen at 20 MHz.

### G. Effects of Out-of-Plane Tissue Motion

**Fig. 10.**
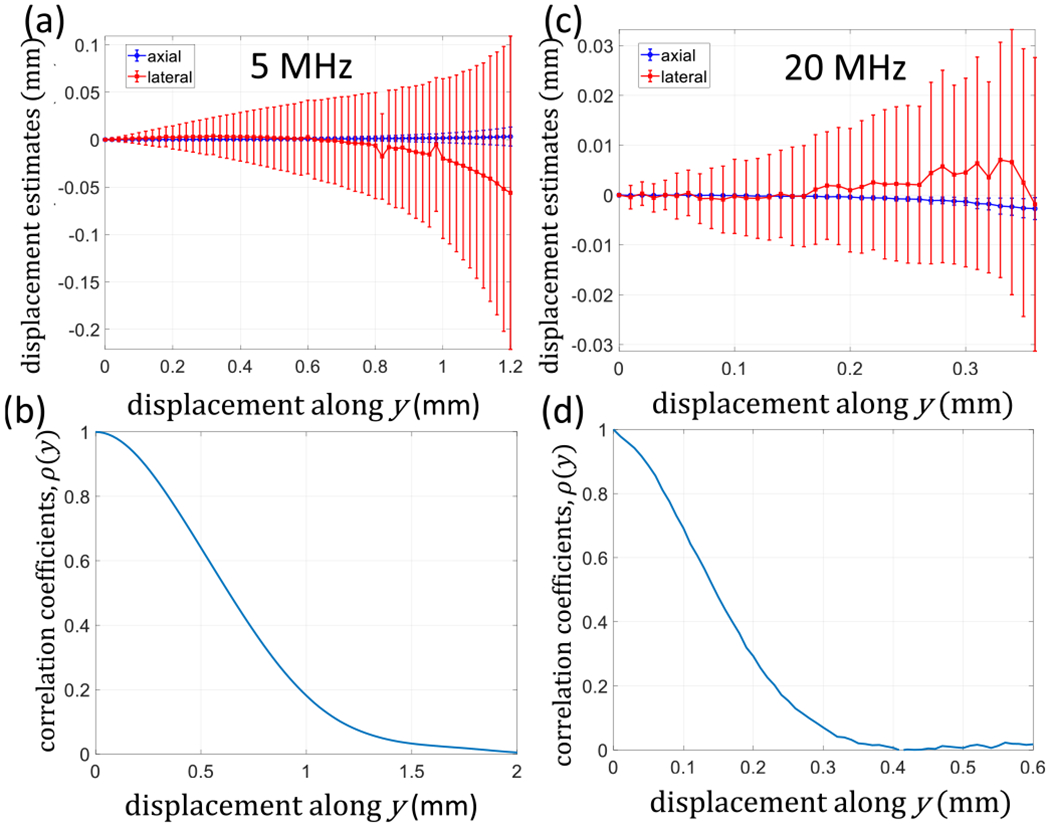
Measurements of in-plane (*x*- and *z*-axes) displacements as a function of out-of-plane (*y*-axis) clutter motion for simulated echo signals. Accurate estimates are obtained when the mean displacement is zero. Measurements are for 5 MHz in (a) and (b) and 20 MHz in (c) and (d). Error bars indicate ±1 sd. The correlation coefficient between each echo frame relative to the first is shown in (b) and (d). The transducer is moved along *y* as echo frames are recorded.

Fig. 10 displays registration displacement errors measured in the *x, z* scan plane as the transducer moves only in elevation (the *y*-axis in Fig. 2) as echo frames are recorded. The echo data are composed of clutter and noise components. We cannot spatially register out-of-plane motion, but we can track its influence on in-plane registration errors as the echo signals decorrelate. Results for 5- and 20-MHz echo simulations are shown in Fig. 10(a) and (c), respectively. The bb simulated pulse properties associated with these results are listed in Table I.

Accurate displacement estimates are zero at all values of y since these simulations contain no in-plane motion. It is clear that the estimation uncertainty increases as the transducer moves in elevation. Also, the errors generated in lateral estimates (red) are much larger than those in axial estimates (blue). Expecting that estimation uncertainty is driven by echo decorrelation, we plotted the interframe echo correlation coefficient as the transducer moves along the *y*-axis. The resulting *ρ*(*y*) curves at 5 MHz are found in Fig. 10(b) and at 20 MHz in Fig. 10(d). The curve shape is determined by the Gaussian-pulse properties: *ρ*(*y*) is four-time broader at 5 MHz than at 20 MHz.

Small translations [see Fig. 10(a) and (c)] along the *y*-axis result in unbiased means and linear increases in the standard deviation of in-plane displacement estimates. Small translations are defined as the range of *y* values where *ρ*(*y*) > 0.5. Greater elevational translations, such that 0.1 *ρ*(*y*) < 0.5, result in nonlinear increases in estimation uncertainties and biased means. At larger translations, where *ρ*(*y*) < 0.1, in-plane estimation breaks down completely. From the curve at 5 MHz, estimates are unbiased for elevational motion < 0.6 mm. At 20 MHz, the limit reduces to 0.15 mm.

Fig. 10 clearly shows that submillimeter elevational motion increases in-plane registration errors. Can this be the reason displacement uncertainties increase with translation distance in the results of Fig. 4? Although the variance bound expressions predict that estimation variance is independent of the true displacement, out-of-plane motion is not considered in (19).

**Fig. 11.**
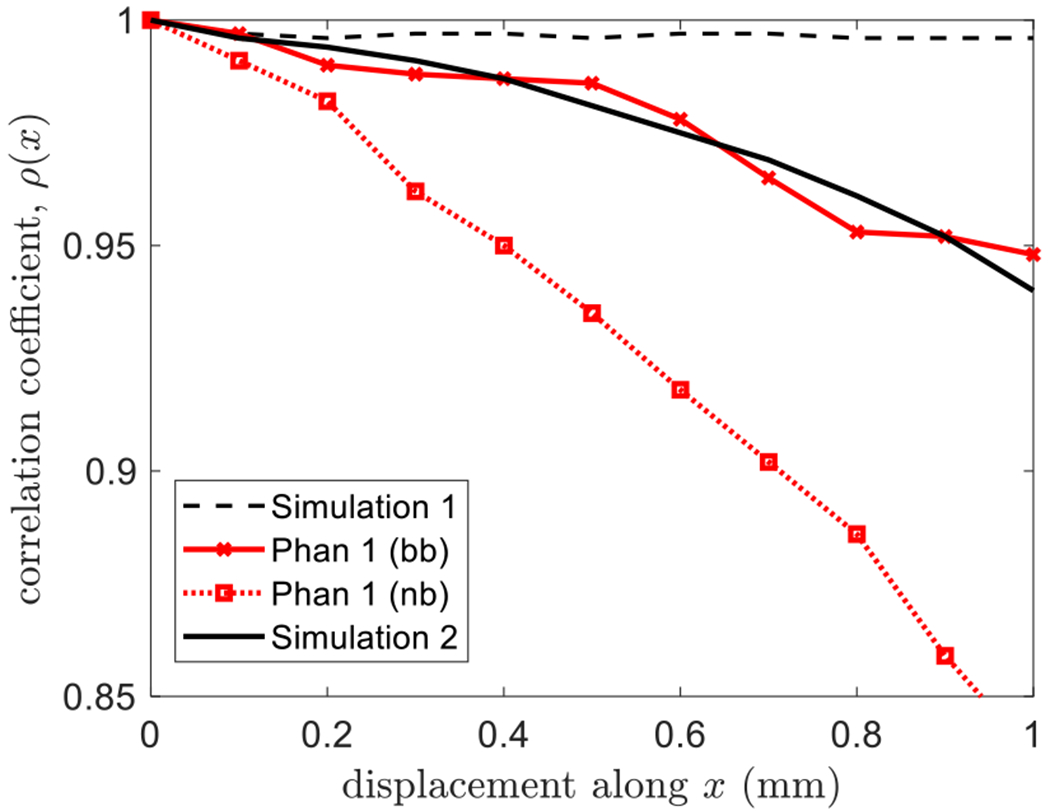
Correlation coefficients are computed from 5-MHz simulated and experimental data. Echo frames that are recorded as the transducer translates along the *x*-axis are spatially registered before *ρ*(*x*) is computed. The dashed and solid black lines are the results from bb echo simulations. The red lines are bb and nb results from phantom experiments.

The amount of out-of-plane clutter motion occurring during lateral translations may be estimated by monitored *ρ* computed after the echo frames are spatially registered. Fig. 11 summarizes the results from phantom experiments (red lines) and echo simulations (black lines). In each case, the transducer is translated with constant speed along the lateral axis *x*, while echo frames are recorded at a constant frame rate.

For echo simulations, transducer motion is exactly coplanar with the scan plane. The resulting interframe correlation coefficient curve (dashed line labeled Simulation 1) shows *ρ*(*x*) ≃ 1 for all *x*. High correlation means that spatial registration was effective at reversing the lateral translation within the limits imposed by noise.

The same lateral translation of the transducer during several phantom experiments always showed some level of decorrelation despite our best efforts to keep the translation in the scan plane. Gross misalignment generates fast decorrelation (not shown). Careful alignment generates the phantom results shown. The nb echo frames recorded during phantom experiments in Fig. 11 decorrelate more quickly than the bb echo frames, as expected from (19).

Assuming that the echo decorrelation *ρ*(*x*) is from simultaneous lateral and elevational motions, we should be able to use *ρ*(*y*) in Fig. 10(b) to determine the amount of motion along the *y*-axis. For example, the bb phantom results in Fig. 11 show that *ρ*(*x*) ≃ 0.95 after the transducer translates laterally *x* = 1 mm. Fig. 10(b) predicts that the transducer must have also moved ~0.15 mm along the *y*-axis. Thus, we simulated the simultaneous transducer motion of *y* = 0.15 mm as *x* = 1.0 mm and found the solid black curve in Fig. 11, labeled Simulation 2. The small elevational movements that increase registration errors also predictably reduce *ρ*. While it is challenging to translate a linear array exactly in its scan plane, the effects of elevational motion on spatial registration are predictable. We find that the most likely reason for an increase in errors with translation distance seen in Fig. 4 is out-of-plane motion.

**Fig. 12.**
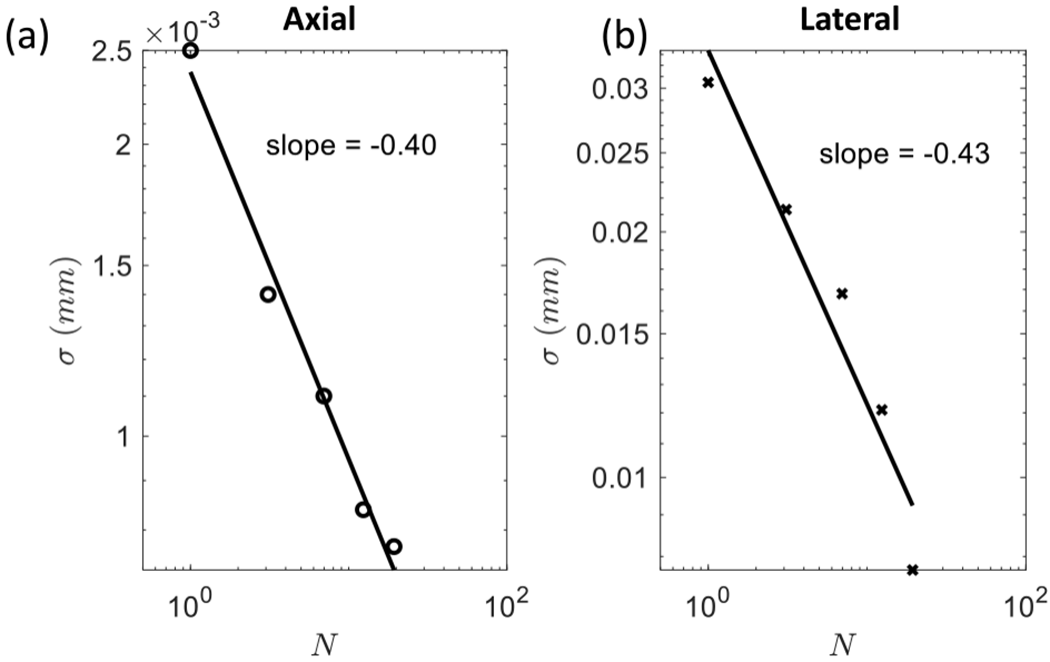
Reduction in standard error for displacement estimates, *τ*, as a function of sample size, *N*. Results for (a) axial and (b) lateral motions are shown. *N* is computed from the sample area used to spatially register frames. Points in each graph, left to right, correspond to sample areas of size 0.5 × 0.5, 1.0 × 1.0, 1.5 × 1.5, 2.0 × 2.0, and 2.5 × 2.5 mm. Lines are linear regression models.

The increase in registration uncertainty caused by elevational motion can be reduced by expanding the area registered. This type of spatial averaging reduces uncertainty by increasing sample size *N*, provided that displacement estimates remain unbiased. To demonstrate, we measured the change in the standard deviation of the mean (i.e., standard error *σ*) for displacement estimates as a function of *N*. The results are shown in Fig. 12. Assuming that the speckle correlation area *A*_2-D_ defines one statistically independent spatial sample in analysis area *X*_0_ × *Z*_0_, then *N* = (*X*_0_*Z*_0_)/*A*_2-D_ ≥ 1 is the number of independent samples. For standard deviation *σ*′, we have $\sigma =\sigma \prime /\sqrt{N}$. The log-log plot of *σ*(*N*) is expected to be a linear curve with slope ln *σ*/1n *N* = −0.5. The slopes estimated from our measurements are closer to −0.4.

## IV. Summary and Discussion

Spatial registration aids power-Doppler clutter filtering by maximizing *ρ* among the echo frames in an ensemble. Accurately registered frames minimize the clutter singular-value bandwidth so that PCA filtering can be effective at eliminating most of the tissue-echo power while minimizing the loss of blood-echo power. Simulation results show that blood-echo power measurements are accurate when the perfusion is directional (see Fig. 6) because the blood singular-value bandwidth is relatively narrow. Clutter filtering is also effective in diffuse perfusion situations, but the blood-echo power estimates are less accurate because the blood and noise subspaces are less distinct (see Fig. 7), making it challenging to select the upper filter threshold outside of the simulation.

The success of rigid spatial registration for improving clutter filtering assumes that: 1) any movement of tissue scatterers is spatially coherent and contained within the *x, z* scan plane;2) the ratio of tissue-to-blood scattering intensity is large; 3) each echo frame may be described as a wide-sense stationary random process over the region used to register frames; and 4) the echo SNR is large. One reason for comparing the results at 20 MHz with those at 5 MHz was to test whether assumptions 2) and 4) hold since both ratios are lower at 20 MHz. We found from the results of Figs. 8–10 that trends observed at 5 MHz are also seen at 20 MHz, except for those that scale with the pulse wavelength. These assumptions generally hold for the conditions commonly encountered in vivo for peripheral perfusion assessments.

A common violation of the four assumptions is out-of-plane clutter motion. Even submillimeter elevational movements will measurably alter the PD-US signal. To minimize clutter motion, we commonly restrain the limb and the probe during data acquisition. Restraints are possible because peripheral perfusion estimates in large muscles are an averaged property.

Figs. 4 and 10 show that the in-plane components of 3-D clutter motion can be registered without bias despite some out-of-plane movement as long as *ρ* > 0.5. The amount of out-of plane motion that might occur can be estimated by monitoring *ρ*. Misregistration errors caused by echo decorrelation are reduced by spatially registering large areas in each frame, provided that the wide-sense stationary assumption holds. We were able to approach the variance bound for phantom measurements reported in Fig. 3 but only for the submillimeter range of motion seen in vivo [see Fig. 5(a)].

The utility of PD-US measurements for patient care depends on how the method fits into the clinical work flow. We see qualitative PD-US methods as monitoring changes in perfusion over time for patients with an established diagnosis. Typically, a PAD patient workup begins with a physical exam and a measurement of the ankle-brachial index (ABI) [41], [42]. If the ABI results are positive for PAD, a CT or MR angiography or vascular ultrasound exam is typically requested to examine arterial patency in the lower extremities. If the conduit vessels are not occluded, a contrast-enhanced quantitative perfusion imaging exam may be conducted [37], [38], [39], [40] to locate regions of low perfusion that could explain symptoms.

One role for PD-US imaging could be to regularly monitor for relative changes in muscle perfusion over a period of weeks and months after a diagnosis is established. The change in perfusion over time might indicate disease progression or treatment responses. This clinical task values measurement consistency over the accuracy, which means that effective clutter filtering is much more important than noise filtering. Since the clutter bandwidth varies significantly depending on the details of tissue motion, setting the clutter-blood threshold on the PCA filter is critical. For simulations and phantoms, it is sufficient to eliminate the first singular value. In PAD patients, the threshold will need to adapt to each patient’s conditions [10], [17]. However, since the acquisition noise is relatively unchanged between patient exams, setting the blood-noise filter threshold must be consistently applied, but the value selected is less critical for qualitative monitoring.

The price paid for using PD-US in place of a contrast-based imaging method is the need to minimize tissue and probe motion, and longer processing time to register echo frames and monitor *ρ*. The benefits of not using contrast-based imaging are the ability to map spatiotemporal changes in perfusion as often as deemed necessary with a modality that is safe, low cost, and widely available.

## Figures and Tables

**TABLE I:** Simulation Parameters for a Gabor Pulse

Independent Parameters	*f*_0_ = 5 MHz	20 MHz

*τ/β*	16 (24.1 dB)	5 (14 dB)
tissue cell # density/blood cell # density	1	1
fast-time sampling rate, 1/*T_k_* (MHz)	38.5	128.3*
axial sampling interval, *cT_k_*/2 (mm)	0.02	0.006
lateral sampling interval, *X* (mm)	0.1	0.06
pulse frequency, *u*_0_ = 2*f*_0_/*c* (mm^−1^)	6.494	25.97
fractional bandwidth (frac BW)	0.75	0.4
in-plane f-number, *z*_0_ / *D_x_*	2	2
elevational-axis f-number, *z*_0_ / *D_y_*	4	4
echo SNR (dB)	30	15
frame-time sampling rate, 1 /*T_m_* (Hz)	10	20
Dependent Parameters	*f*_0_ = 5 MHz	20 MHz

FWHM_*u_z_*_ = *u*_0_ × frac BW (mm^−1^)	4.87	10.39
FWHM_*x*_ = 2×f-number/*u*_0_ (mm)	0.616	0.154
$\sigma_{x}={\mathrm{FWHM}}_{x}/(2\sqrt{2\;\text{ln}\;2})$ (mm)	0.262	0.065
$\sigma_{y}={\mathrm{FWHM}}_{x}/(\sqrt{2\;\text{ln}\;2})$ (mm)	0.524	0.131
$\sigma_{z}=\sqrt{2\;\text{ln}\;2}/(\pi {\mathrm{FWHM}}_{u_{z}})$ (mm)	0.077	0.036
*B_x_* = 1/2*X* (mm^−1^)	5	8.33
*B_z_* = 1/*cT_k_* (mm^−1^)	25	16.7*
*A*_2*D*_ = 4*σ_x_* × 4*σ_z_* (mm^2^)	0.323	0.037
*A*_3*D*_ = *A*_2*D*_ × 4(2*σ_x_*) (mm^3^)	0.677	0.019

* To simulate the process of recording IQ data, we sample the 20 MHz RF echo data at 1/*T_k_* = 128.3 MHz, add acquisition noise, convert data to inphase/quadrature form, low-pass filter and downsample the result by a factor of 5. Consequently, the effective sampling frequency is 25.7 MHz with a temporal (spatial) Nyquist frequency of 12.8 MHz (16.7 mm^−1^).

## Footnotes

1 The backscatter coefficient in whole pig blood (HCT = 40) at 24 MHz is 2 × 10^−3^ cm^−1^sr^−1^ [12]. The backscatter coefficient in rat soleus muscle at normal incidence to muscle fibers is 3.07 × 10^−4^ × *f*^1.61^ = 5 × 10^−2^ cm^−1^ sr^−1^ at 24 MHz [13]. The ratio of muscle to blood backscatter intensity at 24 MHz is 25, with an amplitude ratio of 5. The intensity ratio at 8 MHz is 250 with an amplitude ratio of 16.

2 Estimating SNR experimentally: let $\sigma_{g}^{2}$ and $\sigma_{\in }^{2}$ be the ensemble echo-signal and noise variances. From (18), $\text{SNR}=\sigma_{g}^{2}/\sigma_{\in }^{2}-1≃\sigma_{g}^{2}/\sigma_{\in }^{2}$ for $\sigma_{g}^{2}\gg \sigma_{\in }^{2}$. The noise variance may be estimated for experimental data by subtracting one echo frame from the average of *N* ⪢ 1 echo frames recorded at one spatial location without perfusion. The relationship between the sample and ensemble noise variances is $v\hat{a}r(\epsilon )=\sigma_{\epsilon }^{2}(1-1/\sqrt{N})$. For experimental data, $\text{SNR}≃10\log\left(\sigma_{g}^{2}\left(1-1/\sqrt{N}\right)/\text{v}\hat{\text{a}}\text{r}\left(\in \right)\right)$.
